# Performances of NIPT for copy number variations at different sequencing depths using the semiconductor sequencing platform

**DOI:** 10.1186/s40246-021-00332-5

**Published:** 2021-07-02

**Authors:** Jiexia Yang, Jing Wu, Haishan Peng, Yaping Hou, Fangfang Guo, Dongmei Wang, Haoxin Ouyang, Yixia Wang, Aihua Yin

**Affiliations:** 1 Medical Genetic Centre, Guangdong Women and Children Hospital, Guangzhou, 511400 Guangdong China; 2 Maternal and Children Metabolic-Genetic Key Laboratory, Guangdong Women and Children Hospital, Guangzhou, 511400 Guangdong China; 3 Department of Prenatal Diagnosis Center, Guangdong Women and Children Hospital, No. 521 Xingnan Road, Panyu District, Guangzhou, 511400 China

**Keywords:** Noninvasive prenatal testing (NIPT), Sequencing depth, Copy number variation (CNV), Positive predictive value (PPV)

## Abstract

**Objective:**

To evaluate the performance of noninvasive prenatal testing (NIPT) and NIPT-PLUS for the detection of genome-wide microdeletion and microduplication syndromes (MMSs) at different sequencing depths. The NIPT sequencing depth was 0.15X, and the data volume was 3 million reads; the NIPT-PLUS sequencing depth was 0.4X, and the data volume was 8 million reads.

**Methods:**

A cohort of 50,679 pregnancies was recruited. A total of 42,969 patients opted for NIPT, and 7710 patients opted for NIPT-PLUS. All high-risk cases were advised to undergo invasive prenatal diagnosis and were followed up.

**Results:**

A total of 373 cases had a high risk of a copy number variation (CNV) as predicted by NIPT and NIPT-PLUS: NIPT predicted 250 high-risk CNVs and NIPT-PLUS predicted 123. NIPT-PLUS increased the detection rate by 1.02% (0.58% vs 1.60%, *p* < 0.001). A total of 291 cases accepted noninvasive prenatal diagnosis, with 197 cases of NIPT and 94 cases of NIPT-PLUS. The PPV of CNV > 10 Mb for NIPT-PLUS was significantly higher than that for NIPT (*p* = 0.02). The total PPV of NIPT-PLUS was 12.56% higher than that of NIPT (43.61% vs 30.96%, *p* = 0.03).

**Conclusion:**

NIPT-PLUS had a better performance in detecting CNVs in terms of the total detection rate and total PPV. However, great care must be taken in presenting results and providing appropriate counseling to patients when deeper sequencing is performed in clinical practice.

## Introduction

A copy number variation (CNV) occurs in a segment of DNA with a length of ≥ 1 kbp, and CNVs include insertions, deletions, and duplications, which result in copy number gain or copy number loss [1]. CNVs are important factors affecting human phenotypic variations and diseases [2]. Some CNVs can cause fetal microdeletion and microduplication syndromes (MMSs) that are not related to the age of the pregnant woman [2, 3]. The risk of MMSs in offspring may be higher than that of Down’s syndrome in young women. Studies have shown that the incidence of MMSs in fetuses with normal maternal chromosomes is 1–1.7% [4]. In addition, approximately 12% of cases of unexplained mental retardation, multiple malformations, and stunting are caused by MMSs [5]. Therefore, screening for small chromosome imbalance aberrations during genetic consultations and prenatal diagnoses is of great importance.

There are various methods for the detection of CNVs, including conventional cytogenetic analysis (e.g., G-banded karyotype), microarray-based methods (e.g., comparative genomic hybridization), and next-generation sequencing (NGS). Microarray-based methods are the standard for CNV detection [6, 7]. However, this method has some disadvantages, including its limited resolution and accuracy [8]. Currently, NGS has become a valuable approach for clinical diagnostics in detecting genomic variations with high sensitivity and accuracy.

Noninvasive prenatal testing (NIPT) has been widely adopted for screening common trisomies in obstetric clinical practice for pregnant women, with its higher specificity and greater sensitivity than traditional serum screening [9, 10]. NIPT testing for common aneuploidies has been endorsed by various clinical guidelines for use in high-risk pregnancies [11], and it may expand to average-risk pregnancies in the future. An increasing body of literature is reporting that NIPT is feasible for the detection of autosomal subchromosome fetal abnormalities, such as CNVs [12]. Some studies were performed with a very deep sequencing depth [13], and several studies also evaluated the low-coverage sequencing depth to detect fetal CNVs. Straver et al. reported the detection of large CNVs (over 20 Mb) with a low sequencing depth (0.15–1.66X) [14]. Lo et al. reported an accuracy of 64.5% (20/31) when 4–6 million reads were used to analyze samples with 3 to 42 Mb CNVs [15]. However, there are few research reports comparing CNV detection efficiency using the same platform with different sequencing depths. Thus, we aimed to retrospectively compare the CNV detection efficiency of NIPT using the semiconductor sequencing platform (SSP) with 0.15X and 0.4X sequencing depths. The results are reported below.

## Materials and methods

### Participant recruitment

From July 2016 to December 2019, this retrospective study enrolled women with high-risk pregnancies who underwent NIPT in Guangdong Woman and Children Hospital. The study was approved by the Ethics Committee of Guangdong Women and Children Hospital (number 2013102301). Every participant had accepted detailed genetic counseling and signed a written informed consent form. The inclusion criteria were as follows: (I) isolated advanced maternal age (≥ 35 years, AMA), (II) isolated ultrasound soft-marker abnormality, (III) serological screening for high or intermediate risks, (IV) serological screening for single marker value abnormality (AFP, β-HCG, uE3), and (V) previous adverse outcome of pregnancy or previous pregnancy history of chromosomal abnormalities fetus. Therefore, we divided the pregnant women into 5 groups according to the above inclusion criteria. Pregnant women within the scope of the indication underwent NIPT or NIPT-PLUS according to personal preference.

### Sample preparation and sequencing

Peripheral blood samples (8–10 ml) were withdrawn from the cubital veins of pregnant women, and isolated plasma was centrifuged in an Eppendorf 5810R and 5424 centrifuge (Eppendorf) to obtain cell-free fetal DNA (cffDNA) within 6 h after collection. The samples were stored frozen at − 70 °C as soon as possible until genomic DNA extraction. Then, library construction, quality control, and pooling were performed according to the instructions of JingXin Fetal Chromosome Aneuploidy (T21, T18, and T13) Testing Kits (CFDA registration permit No. 0153400300). Whole-genome sequencing was performed by the semiconductor sequencing technique on the Bioelectronseq 4000 sequencing platform (CFDA registration permit NO. 20153400309). Following DNA library construction, 9~23 libraries were pooled and then sequenced within ~ 200-bp reads, as detailed in our previous article [16]. Fetal DNA concentration was calculated for quality control using our previously described method. Samples that failed to meet the quality criteria, including those for cffDNA extraction, library construction and sequencing, and the fetal DNA concentration (< 4%), were not reported [17]. The NIPT sequencing depth was approximately 0.15X, the data volume was 3 million reads, the NIPT-PLUS sequencing depth was approximately 0.4X, and the data volume was 8 million reads. Combined GC correction and Z-score testing methods were used to identify fetal autosomal aneuploidy of trisomy 21, 18, and 13, as described previously [17]. Additionally, fetal and maternal chromosome copy number variations (CNVs) were classified using our modified Stouffer’s Z-score method as described previously [18]. Simply, each chromosome with an absolute Z-score greater than 3 was marked as having chromosome aneuploidies or microdeletions/microduplications. The purpose of this study was to assess the efficiency of NIPT for CNV detection. Although the aneuploidy of other chromosomes was quantified, it was not the focus of this study.

### Prenatal diagnosis and pregnancy follow-up

Pregnant women who had NIPT results indicating high risk received genetic counseling and were advised to undergo prenatal diagnosis. Chromosomal detection techniques include karyotyping (resolution of G-banding of 400 bands) and chromosome microarray analysis (CMA) (CytoScanTM 750K, available from Affymetrix, USA). To obtain information about neonatal outcomes and newborn growth, we followed up all participants via telephone interviews.

## Statistics

Excel and R language were used for statistical analysis of the data. The positive predictive values (PPVs) of CNVs detected by NIPT were calculated based on prenatal diagnosis results. Fisher’s exact probability tests were used to compare CNV PPVs for NIPT among different groups. The results with *p* values of less than 0.05 were considered statistically significant.

## Results

### Patient characteristics

**Fig. 1:**
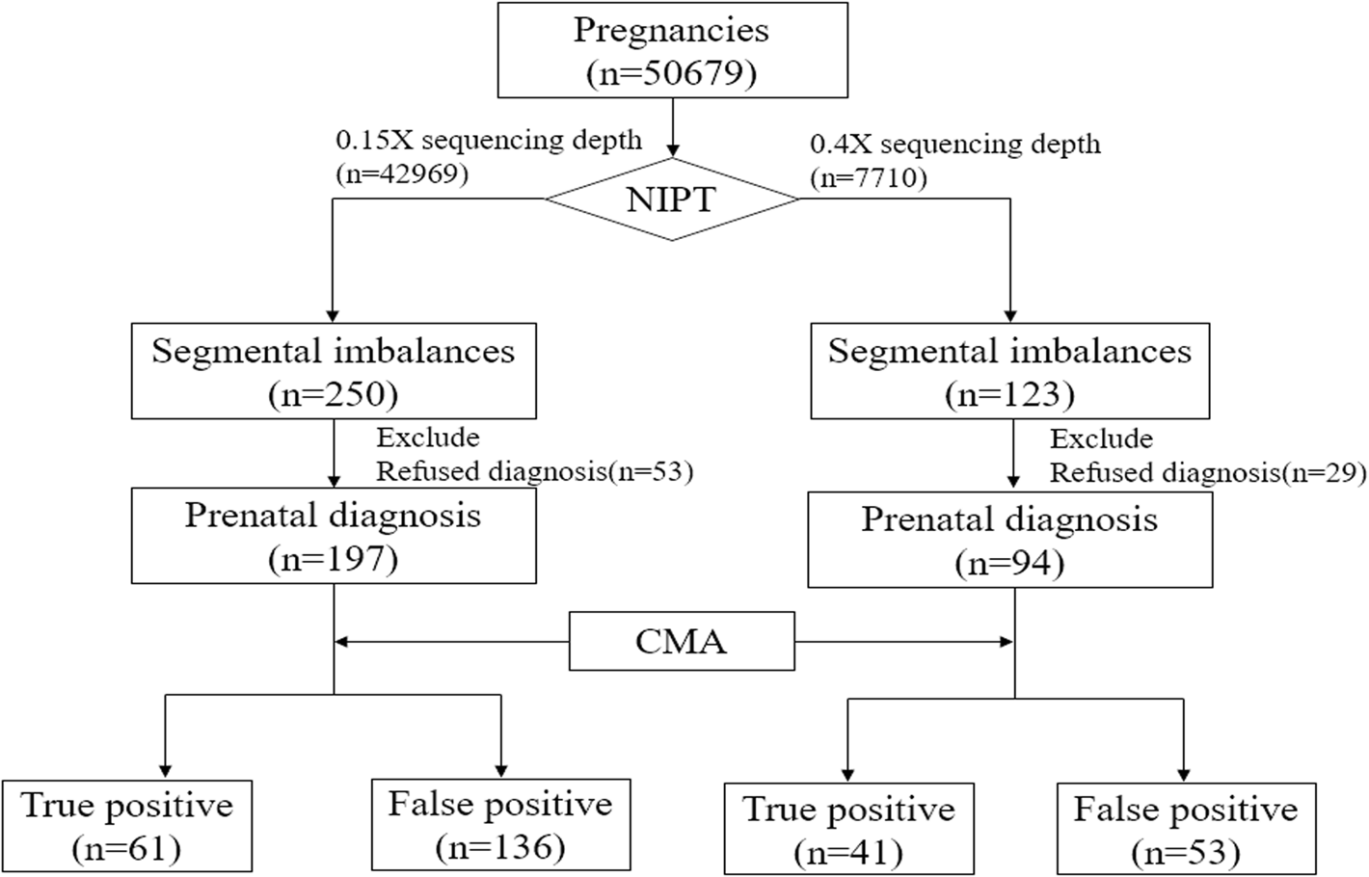
Flowchart of the study

From July 2016 to December 2019, a total of 50,679 patients who met the inclusion criteria were enrolled for NIPT detection in the Prenatal Diagnosis Center of Guangdong Women and Children Hospital. There were 42,969 patients who underwent 0.15X sequencing depth detection, which was NIPT, and the mean age was 31.2 ± 12.8 years. In addition, 7710 patients underwent 0.4X sequencing depth detection, which was NIPT-PLUS, and the mean age was 31.0 ± 10.3 years. The majority (80%) of pregnant women had gestational ages of 12−24 when NIPT was performed, and 29.03% were at high risk by serological screening. Of these 50,679 pregnant women, 6115 were advanced-maternal-age women (AMA, age ≥ 35 years), and 1781 had twin pregnancies. In addition, there were 3246 pregnancies conceived by in vitro fertilization (IVF). Table 1 shows the basic demographic and clinical characteristics, and Fig. 1 shows the study flow of participants.

**Table 1:** Demographic characteristics of the 50679 pregnancies examined by NIPT

Characteristic	NIPT	NIPT-PLUS	*p*
Total	42969 (100.00%)	7710 (100.00%)	
Mean age when performed NIPT (SD), years	31.2 ± 12.8	31.0 ± 10.3	0.07
Singleton pregnancy	41594 (96.8%)	7304 (94.73)	1.2E−19
Twin pregnancy	1375 (3.2%)	406 (5.27%)	1.2E−19
Gestational age at NIPT			
12~19^+6^ weeks	23048 (53.64%)	5005 (67.55%)	3.9E−75
20~23^+6^ weeks	11370 (26.46%)	1565 (21.12%)	3E−30
24~29^+6^ weeks	5036 (11.72%)	824 (11.12%)	0.00903
30~34^+6^ weeks	3279 (7.63%)	311 (4.2%)	8.6E−30
≥ 35 weeks	236 (0.55%)	5 (0.07%)	1.3E−08
Serological screening for high or intermediate risks	20592 (47.92%)	4251 (55.14%)	1.3E−33
Serological screening for the single marker value abnormality (AFP, β-HCG,uE3)	12357 (28.76%)	872 (11.31%)	2E−226
Ultrasound soft-marker abnormalities	4691 (10.92%)	1539 (19.96%)	7E−110
Isolated advanced maternal age (≥ 35 years, AMA)	5094 (11.86%)	995 (12.91%)	0.01
Other^a^	235 (0.55%)	53 (0.69%)	0.13
IVF pregnancies	2535 (5.9%)	711 (9.22%)	5.3E−28
Fetal fragment fraction	13.11%	17.37%	

^a^Previous adverse outcome of pregnancy; previous pregnancy history of chromosomal abnormalities fetus

### Comparison of the detection rates of NIPT and NIPT-PLUS

A total of 373 cases were at high risk of CNVs by NIPT and NIPT-PLUS among 50,679 samples. On the one hand, NIPT predicted 250 high-risk CNVs, including 57 CNVs < 3 Mb, 35 CNVs within 3–5 Mb, 35 CNVs within 5–10 Mb, and 123 CNVs > 10 Mb, which resulted in a total detection rate of 0.58%. On the other hand, NIPT-PLUS predicted 123 high-risk CNVs, including 45 CNVs < 3 Mb, 27 CNVs within 3–5 Mb, 24 CNVs within 5–10 Mb, and 27 CNVs > 10 Mb, which resulted in a total detection rate of 1.60%. NIPT-PLUS increased the detection rate by 1.02% (0.58% vs 1.60%, *p* < 0.001) (Table 2).

**Table 2:** The detection rate of different sequencing depth and CNV size in NIPT and NIPT-PLUS

Index	CNV size	Positive	Positive rate (%)	Total detection rate (%)
NIPT	CNVs (< 3 Mb)	57	0.13	0.58
	CNVs (3–5 Mb)	35	0.08	
	CNVs (5–10 Mb)	35	0.08	
	CNVs (> 10 Mb)	123	0.29	
NIPT-PLUS	CNVs (< 3 Mb)	45	0.58	1.60*
	CNVs (3–5 Mb)	27	0.35	
	CNVs (5–10 Mb)	24	0.31	
	CNVs (> 10 Mb)	27	0.35	

*The total detection rate between NIPT and NIPT-PLUS, *p* < 0.001

### The efficiency comparison between NIPT and NIPT-PLUS

Furthermore, all women who carried a fetus suspected of having a high risk of CNVs were scheduled for a genetic counseling session. During counseling, women were strongly advised to confirm all positive findings by invasive prenatal diagnosis. Thus, a total of 291 cases, including 197 cases of NIPT and 94 cases of NIPT-PLUS, underwent noninvasive prenatal diagnosis (Table 3).

**Table 3:** The efficiency of different sequencing depth and CNV size in NIPT and NIPT-PLUS

Index	CNV size	Prenatal diagnostic validated by CMA			Total PPV
		Positive	Negative	Positive rate (%)	
NIPT	CNV (< 3Mb)	21	21	50.00	61/197(30.96%)
	CNV (3–5Mb)	8	17	32.00	
	CNV (5–10Mb)	12	19	38.71	
	CNV (> 10Mb)	20	79	20.20	
NIPT-PLUS	CNV (< 3Mb)	19	17	52.78	41/94(43.61%)*
	CNV (3–5Mb)	7	10	41.18	
	CNV (5–10Mb)	5	13	27.78	
	CNV (> 10Mb)	10	13	43.48*	

*PPV* positive predictive value

*Significant different between 0.15X and 0.4X sequencing depth

CNVs (> 10Mb) *p* = 0.02

Total PPV *p* = 0.03

The PPV of each CNV size was analyzed. In the NIPT group, the PPV was 50.00% for CNV< 3 Mb, 32.00% for CNV within 3–5 Mb, 38.71% for CNV within 5–10 Mb, and 20.20% for CNV > 10 Mb. In addition, in the NIPT-PLUS group, the PPV was 52.78% for CNV < 3 Mb, 41.18% for CNV within 3–5 Mb, 27.78% for CNV within 5–10 Mb, and 43.48% for CNV > 10 Mb. At the same time, we compared the PPV of the same CNV size in NIPT and NIPT-PLUS. We found that the PPV of CNV > 10 Mb in NIPT-PLUS was significantly higher than that in NIPT (*p* = 0.02). In addition, the total PPV of NIPT-PLUS was 12.65% higher than that of NIPT (43.61% vs 30.96%, *p* = 0.03) (Table 3). Furthermore, when the NIPT and NIPT-PLUS cases were merged into one group, the total PPV for CNVs was 35.05% (102/291), and the incidence of CNVs was 0.57% (291/50679) among pregnant women.

In addition, we compared read numbers between true positive and false positive cases. The average read number of NIPT was 3.57 M, and the read number was not different between NIPT true positive and NIPT false positive cases (*p* = 0.13). The average read number of NIPT-PLUS was 7.60 M, and the read number was also not different between NIPT-PLUS true positive and NIPT-PLUS false positive cases (*p* = 0.76).

### Comparison of PPV for CNV between NIPT and NIPT-PLUS according to different pregnancy characteristics

We divided the pregnancy characteristics of the pregnant women into 5 groups. At the same time, we analyzed and compared the PPVs of NIPT and NIPT-PLUS for different pregnancy characteristics, and there was no difference in PPV for different pregnancy characteristics between NIPT and NIPT-PLUS. Pregnancy with ultrasound soft-marker abnormalities had the highest PPV among the 5 pregnancy characteristic groups for both NIPT and NIPT-PLUS (Table 4).

**Table 4:** Comparison of PPV for CNV between NIPT and NIPT-PLUS according to different pregnancy characteristic

Characteristic	Prenatal diagnostic validated by CMA in NIPT		PPV for CNV in NIPT (%)	Prenatal diagnostic validated by CMA in NIPT-PLUS		PPV for CNV in NIPT-PLUS (%)	*p*
	Positive	Negative		Positive	Negative		
Serological screening for high or intermediate risks	37	70	34.58	16	16	50.00	0.12
Serological screening for the single marker value abnormality (AFP, β-HCG,uE3)	5	20	20.00	7	10	41.18	0.14
Ultrasound soft-marker abnormalities	12	14	46.15	8	5	61.54	0.36
isolated advanced maternal age (≥ 35 years, AMA)	5	28	15.15	6	17	26.09	0.31
Other^a^	2	4	33.33	4	5	44.44	0.67

^a^Previous adverse outcome of pregnancy; previous pregnancy history of chromosomal abnormalities fetus

### CNVs distributed on each chromosome

**Fig. 2:**
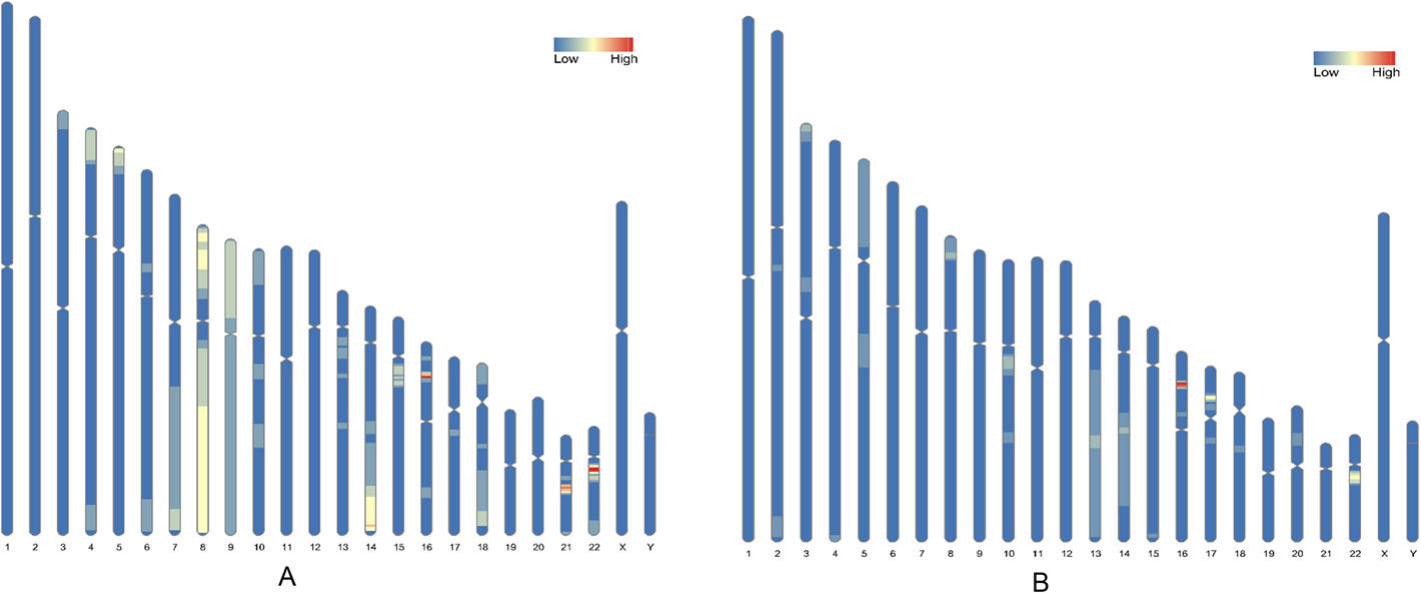
CNVs detected by NIPT and NIPT-PLUS were distributed on each chromosome. **A** NIPT; **B** NIPT-PLUS. Blue indicates that the CNV was not detected. The redder the color, the higher the number of CNVs detected

A total of 61 cases of CNVs were detected by NIPT, including 29 deletions and 36 duplications (4 cases had two CNVs). A total of 41 cases of CNVs were detected by NIPT-PLUS, including 22 deletions and 20 duplications (1 case had two CNVs). CNVs distributed to each autosome are shown in Fig. 2. CNVs on chromosomes 22, 16, 21, and 8 were the most common by NIPT, and CNVs on chromosomes 16, 17, and 22 were the most common by NIPT-PLUS (Fig. 2).

### Pathogenicity classification for true positives detected by NIPT and NIPT-PLUS

According to the “ACMG Genetic Variation Classification Standards and Guidelines” prepared and published by the American Society of Medical Genetics and Genomics (ACMG), the pathogenicity of true positive CNVs was classified. Of the 61 true positives in the NIPT group, 37 (60.66%) were pathogenic, 17 (27.86%) were unknown, and 7 (11.48%) were heredity from phenotypically normal parents. Of the 41 true positives in the NIPT-PLUS group, 23 (56.10%) were pathogenic and 18 (43.90%) were unknown (Table 5).

**Table 5:** Pathogenic classification of the true positive CNVs

Index	n	Pathogenicity	Unknown/VOUS	Heredity from phenotypically normal parents
NIPT	61	37 (60.66%)	17 (27.86%)	7 (11.48%)
NIPT-PLUS	41	23 (56.10%)	18 (43.90%)	0
Total	102	60 (58.82%)	35 (34.31%)	7 (6.87%)

### Follow-up of low-risk pregnancies and women with pregnancies who declined prenatal diagnosis

All cases in this study have been followed up, and all pregnant women have given birth. No visible abnormalities were found in the newborn screening. In addition, 102 patients who refused prenatal diagnosis were followed up, and no visible abnormalities were found in the newborn screening. However, given the special nature of microdeletion and microduplication syndromes, these cases would need to be followed up for a long time.

## Discussion

The present study compared the performance of NIPT and NIPT-PLUS for detecting CNVs, including CNVs of different sizes, detection rates, PPVs, and detected pathogenic fragments, and revealed that NIPT-PLUS had a better performance in detecting CNVs in terms of the total detection rate and total PPV. Furthermore, NIPT-PLUS significantly increased the PPV for CNV > 10 Mb compared with NIPT (*p* = 0.02).

A total of 373 high-risk cases were detected by both NIPT and NIPT-PLUS. NIPT detected 250 high-risk cases in this study, and the total detection rate of NIPT was 0.58%, which was consistent with Hu’s study [19], who reported a detection rate of 0.63% in a cohort of 8141 samples with 51 high-risk CNV results. NIPT-PLUS detected 123 high-risk cases, and the total detection rate of NIPT-PLUS was 1.60% in this study. NIPT-PLUS increased the sequencing depth from 0.15X to 0.4X, which significantly increased the detection rate by 1.02% (*p* < 0.001). Increasing the sequencing depth is likely to reveal more CNVs.

Our results showed that 78.02% (291/373) of pregnant women chose further prenatal diagnosis when CNVs were found by NIPT. Prenatal diagnosis results were considered the “gold standard” for chromosomal diseases. Thus, the PPV of different CNV size groups could be analyzed. The total PPVs of NIPT and NIPT-PLUS for CNVs were 30.96% and 43.61%, respectively. A previous study showed that the PPV for CNVs was 28.99% [12], which was very close to the PPV of NIPT in the present study. Previous clinical validation studies reported variable performance for the detection of specific MMS, and the PPV of NIPT-PLUS in the present study was higher than the previously reported value [12, 20].

A previous study showed that CNV size seemed to be a major determinant of the performance of NIPT, meaning NIPT was better at predicting large segment abnormalities [17]. Liang [20] reported that CNVs ≥ 10 Mb (PPV 32%) were much higher than CNVs < 10 Mb (PPV 19%). Li reported that CNVs > 5 Mb could be detected with high sensitivity (90.9%), whereas CNVs < 5 Mb had reduced sensitivity [21]. An interesting point was that the PPV of CNV<3 Mb was the highest for both NIPT and NIPT-PLUS in the present study, which was different from previous reports. The reason may be that the majority of cases with CNV < 3 had pathogenic variants in our study. For example, there were 12 pathogenic CNVs in the CNV < 3 Mb NIPT group; among them, 5 cases had CNV at 22q11.2. 22q11.2 deletion syndrome is the most common microdeletion syndrome, with an estimated prevalence of 1:3000 to 1:6000 children and 1:1000 unselected fetuses [4, 22–24]. This condition is characterized by congenital heart disease (especially conotruncal defects), immunodeficiency, hypoparathyroidism, palatal, gastrointestinal, skeletal and renal abnormalities, characteristic facial features, developmental and speech delay, and an increased risk for psychiatric illness; early recognition is imperative [24, 25]. Thus, NIPT testing tends to select CNVs with high incidence and more significant signals to report.

In addition, the total PPV of NIPT-PLUS was higher than that of NIPT, and the PPV of NIPT-PLUS was increased by almost 13% compared with NIPT (*p* = 0.03). Therefore, increasing the sequencing depth improved not only the detection rate improved but also the PPV. Our previous study showed that when the sequencing depth was raised from 3.5 million reads to 10 million reads, the detection rate was raised, and the sensitivity improved from 69 to 73% [17].

In addition, we compared the PPVs of NIPT and NIPT-PLUS for different CNV sizes. The CNV size seemed to be the major determinant of the performance of this SSP method. We found that when the CNV size was > 10 Mb, the PPV significantly increased with NIPT-PLUS (from 20.20 to 43.48%, *p* = 0.02). Increasing the sequencing depth is likely the most direct way to improve the diagnostic accuracy in general and is increasingly likely to be realized with additional reductions in sequencing costs.

There are some tendencies among pregnant women who choose either NIPT or NIPT-PLUS. We compared the PPVs for CNVs of NIPT and NIPT-PLUS according to different pregnancy characteristics. We found that pregnancies with ultrasound soft-marker abnormalities and serological screening indicating high or intermediate risk had higher PPVs with both NIPT and NIPT-PLUS than the other 3 groups of women, but there was no difference between the NIPT and NIPT-PLUS groups according to pregnancy characteristics. In a previous study, Chen also compared PPVs for CNVs and showed ultrasound marker abnormalities and high-risk by serological screening as being associated with a higher PPV [12]. Since pregnant women chose NIPT or NIPT-PLUS nonrandomly, some deviations may be introduced in the prediction rate estimation, which was a limitation of the present study. Therefore, a large-scale NIPT study of different pregnancy characteristics is a direction of our further research.

In addition, there was one interesting finding, namely, that there was a difference in the CNV distribution between NIPT and NIPT-PLUS (Fig. 2). Regarding the characteristics of the pregnant women, we found that pregnant women with ultrasound soft-marker abnormalities were more inclined to choose NIPT-PLUS (11.07% vs 20.1%). Soft markers found during the second-trimester ultrasound imaging can include nuchal thickening, an echogenic cardiac focus or foci, an echogenic bowel, pyelectasis, choroid plexus cysts, a shortened femur or humerus, an absent nasal bone, and a single umbilical artery [26]. These markers may, however, be associated with an increased risk for fetal chromosomal abnormalities [27]. Thus, soft-marker differences may result in differences in CNV distribution between NIPT and NIPT-PLUS.

One crucial challenge is that increasing the sequencing depth may identify more deletions and duplications of unknown clinical significance, with incomplete penetrance or with mild or unpredictable clinical consequences. Many of these abnormalities may be normal inherited variants. For example, we found 35 cases of unknown and 7 cases of heredity from phenotypically normal parents in the present study. Thus, great care must be taken in presenting results and providing appropriate counseling to patients when deeper sequencing is performed in clinical practice. We expect that the utility of the deeper sequencing depth of NIPT for subchromosomal abnormalities will increase with greater understanding of genomic disorders.

## Conclusion

In conclusion, this study compared two sequencing depths with SSP. NIPT-PLUS had a better performance in detecting CNVs in terms of the total detection rate and total PPV, and the PPV of NIPT-PLUS for CNV > 10 Mb was significantly increased compared with that of NIPT. However, one crucial challenge is that an increased sequencing depth may identify more deletions and duplications of unknown clinical significance. Thus, great care must be taken in presenting results and providing appropriate counseling to patients when deeper sequencing is performed in clinical practice.

## Data Availability

The datasets used and/or analyzed during the current study are available from the corresponding author on reasonable request.

## Abbreviations

NIPT: Noninvasive prenatal testing
PPV: Positive predictive value
CNV: Copy number variation
cffDNA: Cell-free fetal DNA
ACMG: American Society of Medical Genetics and Genomics
CMA: Chromosome microarray analysis
MMS: Microdeletion and microduplication syndrome
NGS: Next-generation sequencing
SSP: Semiconductor sequencing platform
